# Integrating stent design and microstructural characterization to improve clinical outcomes of bioresorbable stents

**DOI:** 10.1016/j.matdes.2025.115013

**Published:** 2025-10-30

**Authors:** Francesc Canalejo-Codina, Marta Pegueroles, Andrés A. García-Granada, Jordi Martorell, Elazer R. Edelman, Mercedes Balcells

**Affiliations:** a Institute for Medical Engineering and Science, Massachusetts Institute of Technology, Cambridge, MA, USA; b Vascular Engineering and Applied Biomedicine Group (GEVAB), IQS School of Engineering, Universitat Ramon Llull, Barcelona, Spain; c Industrial Product Engineering Group (GEPI), IQS School of Engineering, Universitat Ramon Llull, Barcelona, Spain; d Biomaterials, Biomechanics and Tissue Engineering Group, Department of Materials Science and Engineering, Center for Research in Multiscale Science and Engineering (CCEM) and Institute for Research and Innovation in Health (IRIS), Universitat Politècnica de Catalunya, EEBE, Barcelona, Spain

**Keywords:** Bioresorbable stent, Design, Manufacturing, Microstructure, Degradation, Thrombosis

## Abstract

Bioresorbable stents were conceived to revolutionize the treatment of cardiovascular diseases. However, their significant benefits were overshadowed by a higher clotting rate compared to permanent implants. This clinical failure is linked to strain-induced microstructural disruptions during fabrication and implantation, resulting in heterogeneous loss of structural integrity. The non-gradual loss of support, combined with faster, localized polymer deterioration, directly contributes to the clinical failure observed in bioresorbable stents. Leveraging this understanding marks a significant advancement toward their safe reintroduction. However, the extent to which a stent’s stress distribution interacts with the polymer’s microstructure remains understudied.

This study advances the existing knowledge on bioresorbable stents by establishing a framework for comprehending the microstructural properties that emerge from stent fabrication and implantation, ultimately aiming to improve clinical outcomes. The analysis addresses structural degradation and thrombogenicity of the devices, linking these aspects to the microstructural characteristics of various poly(L-lactide-co-ε-caprolactone) stent configurations. The configuration with the polymer microstructure tailored to the stress profile of the stent design presented the best performance. These findings emphasize the critical need to align the as-manufactured material properties with the stress distribution during implantation and provide powerful tools and strategies to cast bioresorbable stents that outperform current cardiovascular stents.

## 1. Introduction

Despite recent advancements in interventional vascular procedures [1–5], cardiovascular diseases remain the leading cause of death worldwide [6–8] and a major contributor to hospitalizations [9]. Since the widespread adoption of bare metal stents (BMS) in the 1980s and drug-eluting stents (DES) in the early 2000s, these devices have become essential for revascularizing patients with symptomatic artery disease [10,11]. By providing stenting to the vessel wall, stents prevent acute and subacute vessel closure and constrictive remodeling [12].

Bioresorbable stents (BRS) are designed to provide temporary support to narrowed vessels, promoting healthy remodeling after intervention [5,13,14]. Ideally, the stent’s mechanical support gradually diminishes as it degrades, eventually disappearing [15,16], and potentially avoiding the long-term complications associated with permanent implants [17]. However, current BRS have a significantly higher risk of thrombosis and myocardial infarction [18–20], highlighting the need for new designs that address these limitations [21].

Semicrystalline polymers like poly(L-lactide-co-ε-caprolactone) (PLCL) exhibit a unique microstructure consisting of crystalline domains with differentially aligned molecules, and amorphous regions with randomly oriented molecular arrangement. These microstructures strongly influence macroscopic properties, such as mechanical strength and degradation rate [22–24]. Therefore, characterizing the stent’s microstructural ordering, along with its thermally and mechanically strain-induced heterogeneity during manufacturing and implantation, is crucial for predicting their clinical performance [25,26].

Localized disruptions in microstructural ordering can accelerate stent degradation, leading to heterogeneous loss of structural integrity [27]. This effect is intensified by the dynamic environment stents operate in, where cyclic stresses from stretching, torsion, and bending pose additional challenges [17,28]. Non-gradual, uneven loss of structural support directly contributes to acute and subacute clinical failure of the device [26,29]. Loss of lumen patency, stent recoil, and malap-position in specific regions significantly disrupt arterial remodeling, elevating the risk of thrombosis and myocardial infarction [30–32]. Leveraging this understanding marks a significant step toward the safe clinical reintroduction of BRS.

To date, no study has examined how variations in microstructural ordering of the base material modulate stress-induced disruption in BRS. Specifically, the extent to which a stent’s stress distribution interacts with—and is amplified or mitigated by—the polymer’s alignment and crystallinity remains entirely unquantified. This is of outmost importance to define design principles for polymeric stents that integrate microstructural ordering with device-specific stress profiles.

This study builds upon the current state of the art to establish a framework for comprehending the microstructural properties of BRS. The research conducted in this series of sequential experiments was driven by the hypothesis that a polymeric construct with microstructural characteristics tailored to the stress distribution of the stent design could minimize the strain-induced heterogeneity of the stent’s material, thereby improving the clinical outcomes of the device.

An analysis was conducted to evaluate four PLCL stent configurations, combining two as-manufactured material properties with two implantation-related stress distributions. Microstructural ordering was assessed before and after implantation into silastic tubes. Implanted BRS underwent accelerated thermal degradation at 48 °C in PBS for 0, 15, 30, and 45 days, then were tested in a modified Chandler loop system with human blood and physiological flow. Structural degradation and thrombogenicity were analyzed and correlated with microstructural features. The study established the link between material tubing, stent design, and *in vitro* biological performance. The configuration with a microstructure tailored to a balanced stress distribution showed the best performance, underscoring the need to align material properties with implantation stresses to preserve microstructural integrity and improve clinical outcomes.

## 2. Materials and methods

The tubing material was Purasorb^®^ PLC 9538 (Corbion, Netherlands), a 95:5 poly(L-lactide):poly-ε-caprolactone (PLLA:PCL) copolymer with a viscosity of 3.8 dl/g, molecular weight of 700,000 g/mol, glass transition temperature of 55 °C, and melting temperature of 180 °C. The extruded construct, Absorv^™^ PLC 95L/5C Extruded Special XSE (Zeus Inc., USA), had a diameter of 7.493 mm and wall thickness of 127 μm. Tubes were laser-cut into stents using a femtosecond laser (MeKo Manufacturing e.K., Germany). All stents were produced using identical laser-cutting parameters to minimize differences in laser-engraved surface roughness.

Material properties of polymer tubing were assessed based on tensile tests of miniature dog-bone specimens. According to ASTM D882–18, a total of 3 specimens per type were clamped between two toothed hydraulic grippers with a clamping length at the start position of 10 mm. Then, a calibrated load cell of 50 lbf, a preload of 500 mN, and a test speed of 2.5 mm/min were used to pull the specimens.

Stent configurations were varied by iteratively adjusting the tubing extrusion and expansion parameters (strain rate, expansion outreach, temperature gradient) according to the methods described by Lindsey et al. in the vendor’s (Zeus Inc., USA) patent [33] and design features (rounding radii, strut width, angle, curvature) according to the simulation model later introduced. As-manufactured stents were imaged at 10 × magnification using a Leica M165 C optical microscope (Leica Microsystems, Germany).

**Fig. 1.**
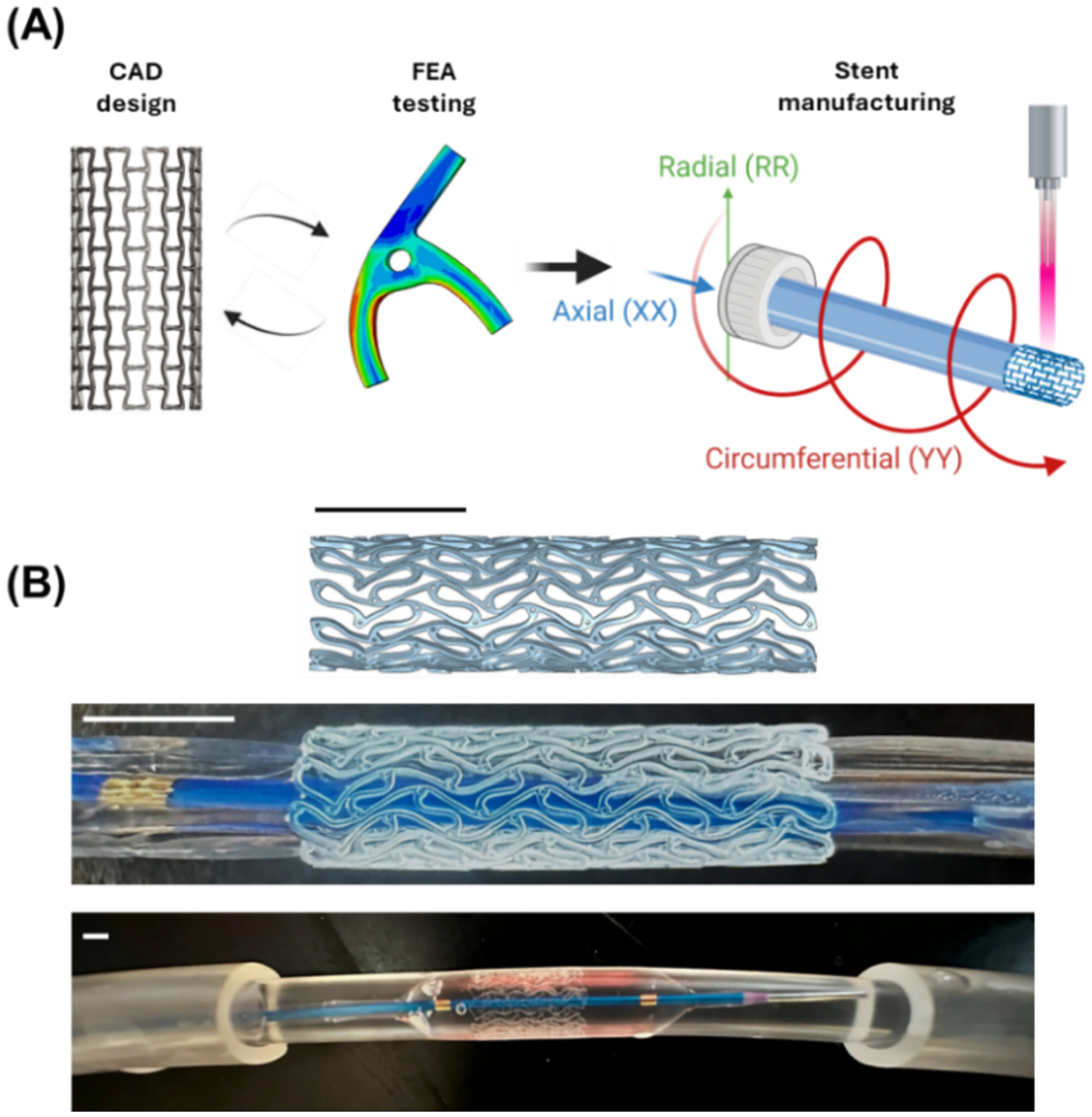
(A) Stent design via CAD modeling and FEA simulation, followed by tube extrusion and laser cutting. (B) Stent implantation process: computational crimping, experimental crimping, and balloon expansion (top to bottom). Scale bar = 2 mm.

The crimping and expansion of PLCL stents followed a controlled, multi-stage protocol. During crimping, the stent, guidewire (V-18, Boston Scientific), balloon catheter (Sterling Monorail 5.0 × 20 × 135 mm), and RSS Crimping Tool (Blockwise Engineering) were placed in a 48 °C deionized water bath. The stent was gradually compressed at 1 mm/min to a 2.0 mm diameter. For expansion, the assembly was placed in a 37 °C water bath inside silastic tubing. The balloon was inflated to nominal pressure for 30 s, deflated, and held for another 30 s, as shown in Fig. 1.

### 2.1. Finite Element analysis

An explicit Finite Element Analysis (FEA) (Abaqus, Dassault Systèmes, France) was performed to assess stress and strain distribution in stent designs during crimping with radially oriented plates. Stent geometries were created in SolidWorks (Dassault Systèmes, France), and material properties were assigned based on tensile tests. A full 3D model of the stents’ geometry, without spatial simplifications, was used to accurately replicate experimental conditions [34].

### 2.2. Raman spectroscopy

**Fig. 2.**
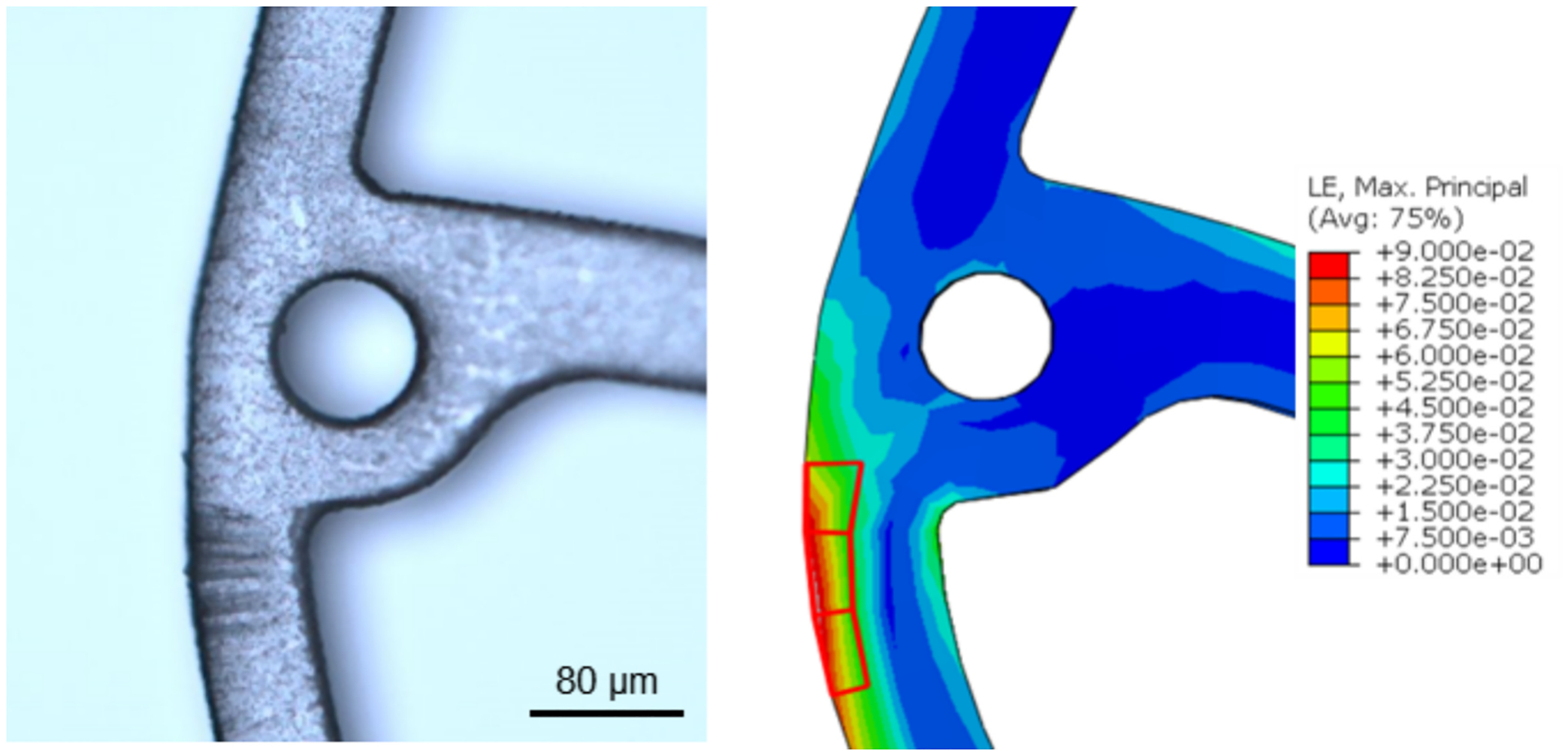
Confocal microscopy image and computational analysis of Von Mises stress distribution [MPa] of peak feature regions of the stent.

A Raman Reflex spectrometer (Renishaw, UK) and 532 nm argon laser was used to analyze stent surfaces with a spatial resolution of 1.0 μm and detection depth of 0.7 μm. Raman scatter was collected in backscattering geometry through a 100 × objective and recorded with a CCD camera. Spectra were acquired from 500–2000 cm^−1^ using a 1200 l/mm grating (633/780), standard scan type, and confocal setup. Acquisition parameters included a 4 s exposure, 100 % laser power, and 4 accumulations. Non-polarized and polarized Raman spectroscopy were performed on the outer surface (OS, in contact with vessel walls), inner surface (IS, in contact with luminal flow), and Core (the interior cross-section) of the stents. Samples were cut to expose the core and aligned with the X polarization axis. Measurements were taken before and after implantation in high-stress regions identified during crimping and expansion, as observed in Fig. 2.

Material crystallinity was quantified using non-polarized Raman spectroscopy. Polymer chain orientation was assessed using polarized Raman spectroscopy with two polarization geometries, Z(XX)Z and Z (YY)Z, based on Porto’s notation [35]. This resulted in four different measurements: neutral (N, non-polarized), radial (RR, polarized), circumferential (XX, polarized) and axial (YY, polarized). Spectral analysis was performed with WiRE^™^ 5.2 (Renishaw, UK) and Matlab R2023b (MathWorks, USA), applying baseline subtraction with a third-degree polynomial and smoothing with a second-degree polynomial over a 7 cm^−1^ window.

I_875_ was calculated by integrating from 825 to 900 cm^−1^, representing the stretch mode of the polymer backbone. I_1452_ was calculated by integrating from 1,425 to 1,500 cm^−1^, reflecting the asymmetric bending mode of CH3. I_925_ was calculated by integrating from 910 to 930 cm^−1^, representing the CH_3_ rocking mode mixed with the stretch mode of the backbone [36,37].

Polarized Raman spectroscopy used the **I**_**875**_**/I**_**1452**_ **intensity ratio** as a marker of molecular orientation [26]. The **I**_**875**_**/I**_**1452 – YY/XX**_ **polymer orientation ratio** was calculated by dividing this ratio in the Z(YY)Z and Z(XX)Z polarization geometries, providing a quantitative measure of polymer chain alignment: values greater than 1 indicate predominant circumferential alignment, values equal to 1 indicate an even distribution between circumferential and axial directions, and values less than 1 indicate predominant axial alignment. The **I**_**875**_**/I**_**1452 – YY/XX – Post-expanded/Pre-crimped**_ **polymer orientation impact ratio** compared orientation ratios before and after implantation to assess implantation impact on polymer alignment: a value greater than 1 signifies a shift toward circumferential alignment, less than 1 a shift toward axial alignment, and equal to 1 no change.

Non-polarized Raman spectroscopy used the **I**_**925**_**/I**_**1452**_ **intensity ratio** as a marker of crystallinity [26]. The **I**_**925**_**/I**_**1452 – Surface/Core**_ **polymer crystallinity ratio** compared crystallinity between the stent surfaces and core: values greater than 1 indicate higher surface crystallinity, equal to 1 indicate equal crystallinity, and less than 1 indicate higher core crystallinity. The **I**_**925**_**/I**_**1452 – Surface/Core – Post-expanded/Pre-crimped**_ **polymer crystallinity impact ratio** assessed changes in crystallinity distribution caused by implantation: values greater than 1 indicate increased surface crystallinity relative to the core, less than 1 indicate increased core crystallinity, and equal to 1 indicate no change.

A detailed schematic representation of the Raman spectroscopy data analysis process and ratio calculations can be found in Supplementary Fig. S1.

### 2.3. Degradation and thrombogenicity test on stents

Four stents per PLCL stent configuration were implanted into 24 mm-long segments of C-Flex^®^ Transfer Tubing (3/16″ ID × 1/4″ OD; Masterflex, Cole Parmer, Avantor), following the standardized implantation protocol. Each implanted tube was filled with 8 mL of phosphate-buffered saline (PBS) using a 10 mL Luer-lock syringe (Merck KGaA, Germany) and incubated at 50 °C with 5% CO_2_. PBS pH was monitored throughout using an Expert-Pro ISM pH meter (Mettler Toledo, Spain). Stents were retrieved at 0, 15, 30, and 45 days of accelerated thermal degradation for subsequent thrombogenicity testing.

A modified Chandler loop system was used to assess thrombosis in the endovascular devices [38]. Testing loops were formed by converting the straight, stent-implanted segments into closed rings, as observed in Fig. 4. The ends were joined using a 1.5 cm segment of C-Flex^®^ Transfer Tubing (1/4″ ID × 3/8″ OD; Masterflex, Cole Parmer, Avantor). A 1 cm elastic band of C-Flex^®^ Transfer Tubing (3/8″ ID × 1/2″ OD) was placed coaxially over the junction to provide radial compression and additional support.

Human blood was collected following institutional protocols (Research Blood Components, USA) using a 10% acid-citrate-dextrose (ACD) solution (85 mmol/L trisodium citrate, 69 mmol/L citric acid, 111 mmol/L glucose; pH 4.6) to prevent clotting. Immediately before testing, blood was repleted with a CaCl_2_/MgCl_2_ solution (100 mmol/L CaCl_2_, 75 mmol/L MgCl_2_) at 62.5 μL per 1 mL of blood to neutralize the anticoagulant effects of ACD.

**Fig. 3.**
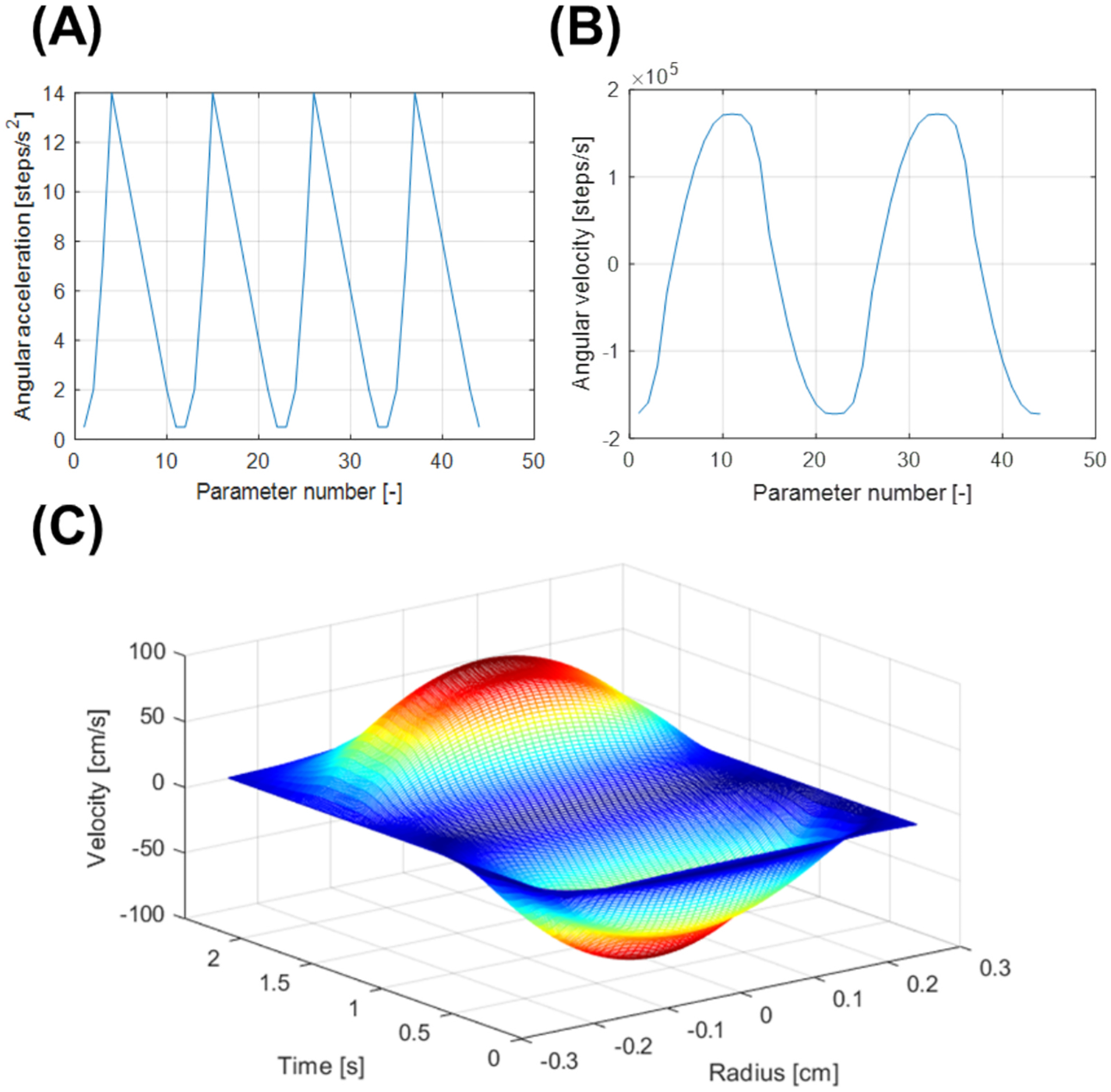
Pulsatile flow simulating physiological arterial hemodynamics in accelerated blood-filled loops. (A) Angular acceleration of motor-controlled rotor. (B) Angular velocity of motor-controlled rotor. (C) Pulsatile, parabolic flow profile as a function of silastic tubing radius for a complete rotation cycle.

**Fig. 4.**
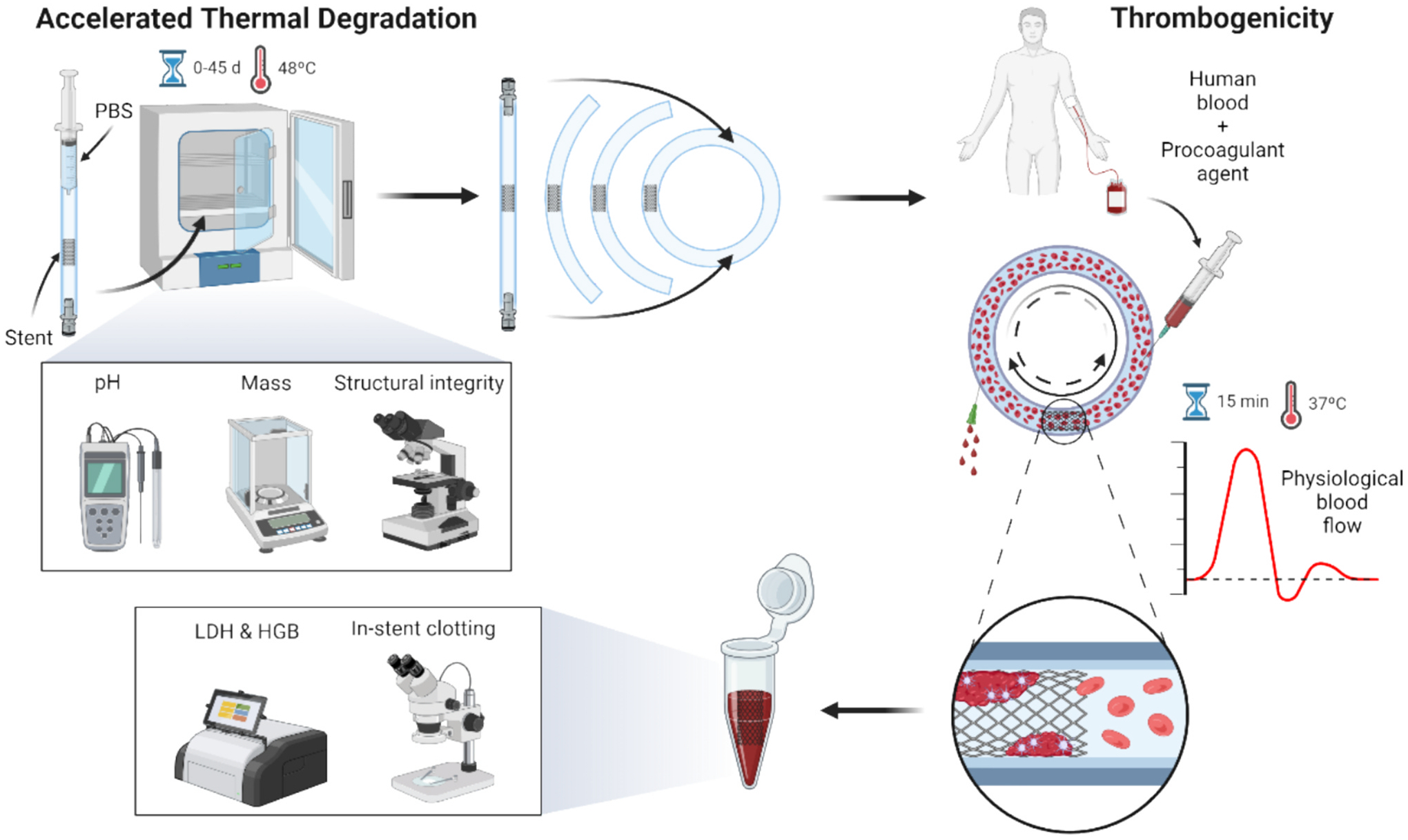
Accelerated thermal degradation and thrombogenicity testing. Stent-implanted segments were incubated at 48 °C for up to 45 days to induce PLCL degradation, then tested under physiological flow with human blood.

Flow loops were filled with blood to ensure complete air displacement. A 23G dermal needle was used for blood infusion—large enough to prevent cell damage yet small enough to avoid air entry—while an 18G needle allowed air to escape. After filling, the rotor was mounted, and the system was run for 15 min to promote in-stent clot formation. Motor-controlled rotors generated pulsatile flow simulating physiological arterial hemodynamics (average flow rate: 185 mL/min) [38]. Flow rates were recorded and validated using a Transonic PXL Clamp-On Flow Sensor and T106 Flowmeter (ADInstruments, Australia), a National Instruments LAB-PC A/D interface (USA), and LABTECH software (ConnectWise, USA), as observed in Fig. 3.

After testing, free blood was removed, and the stented segments were flushed with 60 mL of Tyrode solution supplemented with HEPES (0.01 mol/L) and MgCl_2_ (0.75 mmol/L) at a constant rate of 0.2 mL/s. Adherent clot was assessed visually (wall-associated clot) and biochemically. Following visual inspection and imaging, segments were excised and incubated with 0.5 mL of 1% Triton X solution for 20 min to induce clot lysis. Equivolume lysates were collected for biochemical analysis: lactate dehydrogenase (LDH, total cellular content; Cyto-Tox 96 Assay, Promega) and hemoglobin (HGB, red blood cell content; absorbance at 405 nm) were measured to quantify thrombogenicity. The HGB/LDH ratio was used as a normalized metric to compare thrombogenic potential across samples.

Stents were rinsed with deionized water, dried at 37 °C for 24 h, and stored in a desiccator until further analysis. Post-retrieval mass was recorded and compared to initial mass to evaluate degradation. Structural integrity was assessed visually, and degraded stents were imaged at 4 × and 10 × magnification using a Nikon Eclipse Ti confocal microscope (Nikon, Japan) with a SPOT Idea 29.2 camera (SPOT Imaging, USA) in brightfield mode, under temperature-controlled conditions (24 ± 2 °C).

BRS results were compared against four non-stented flow loops (negative controls), and two metallic stent references: four Cordis Palmaz Genesis 6 × 12 mm stents (Cordis, USA) and four Cook Medical Formula 418 7 × 12 mm stents (Cook Medical, USA).

### 2.4. Statistical analyses

Data are presented as mean ± SD unless otherwise specified. Sample sizes were n = 6 for non-polarized and polarized Raman spectroscopy, and n = 4 for each degradation timepoint and thrombogenicity run. One-tailed Student’s t-tests with unequal variance were conducted for microstructural characterization results, and two-tailed Student’s t-tests with unequal variance were conducted for *in vitro* results. A P value < 0.05 was considered to denote statistical significance (* for P < 0.05, ** for P < 0.01, *** for P < 0.001).

## 3. Results

### 3.1. Macro- and Microscopic characterization of stent configurations

PLCL stents were manufactured through a process of extrusion and laser cutting. The tubing process enabled precise control over the PLCL material’s micro- and macroscopic properties, while ultra-high frequency femtosecond laser cutting ensured high-quality stent fabrication, as observed in Fig. 6B.

**Fig. 5.**
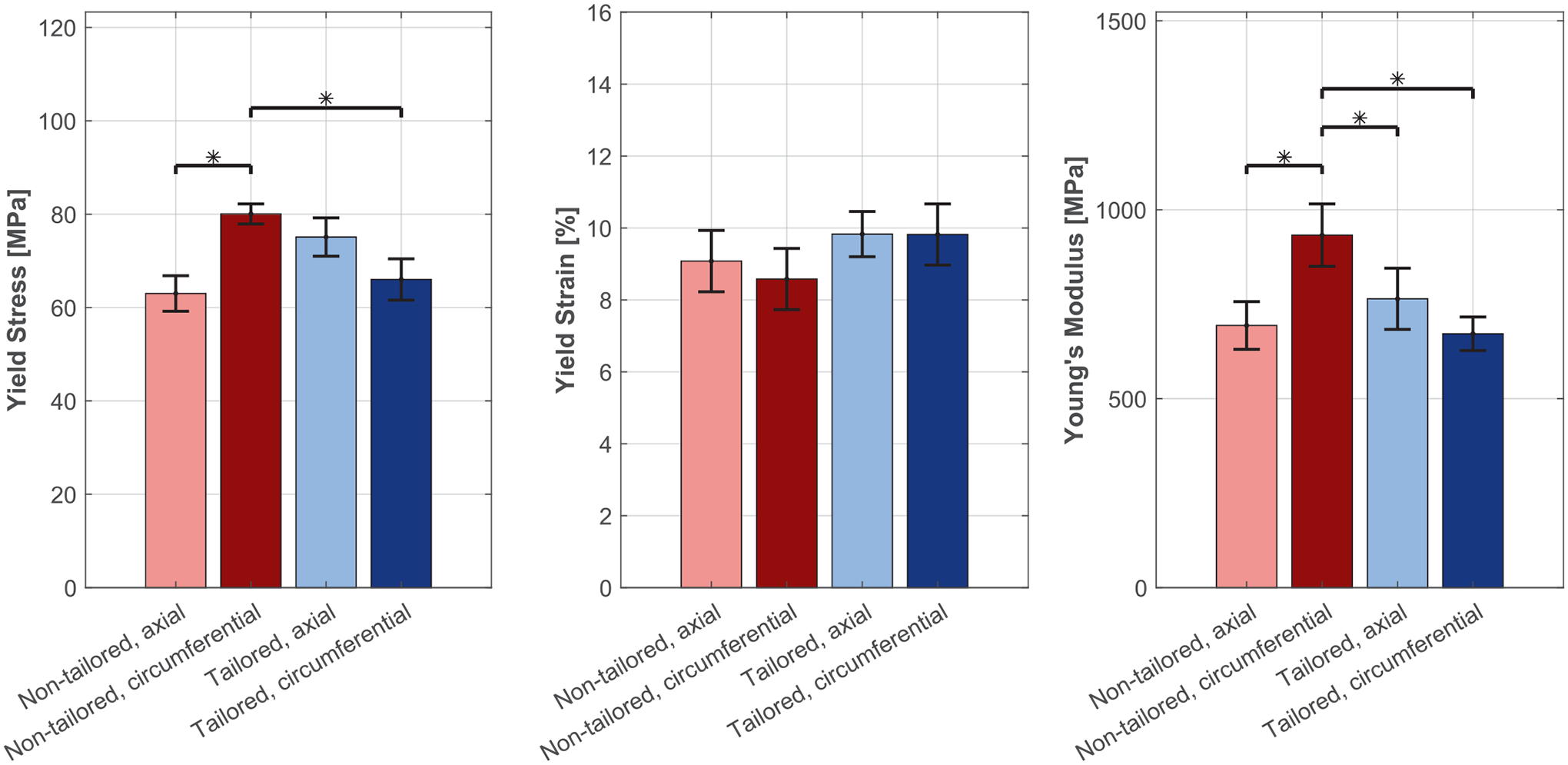
Mechanical properties (Yield Stress, Yield Strain, and Young’s Modulus) for the non-tailored and tailored tubing formations.

Two tubing types were developed: (I) non-tailored and (II) tailored. The non-tailored tubing exhibited enhanced circumferential polymer chain orientation, increasing hoop strength—critical for radial vessel support—but lacked uniform crystallinity across the wall thickness. In contrast, the tailored tubing showed balanced chain alignment between axial and circumferential directions, designed to match uniform implantation stresses and achieve homogeneous crystallinity throughout the tube. These microstructural differences were reflected in the mechanical properties reported in Fig. 5.

The computational simulation model produced two distinct stent designs: (I) non-balanced and (II) balanced. The non-balanced design exhibited a one-phase stress profile, in which the vertical struts always carried higher stress than the horizontal struts. In contrast, the balanced design featured a two-phase stress distribution: vertical struts initially carried the highest stress, which then shifted to horizontal struts as the diameter decreased.

**Fig. 6.**
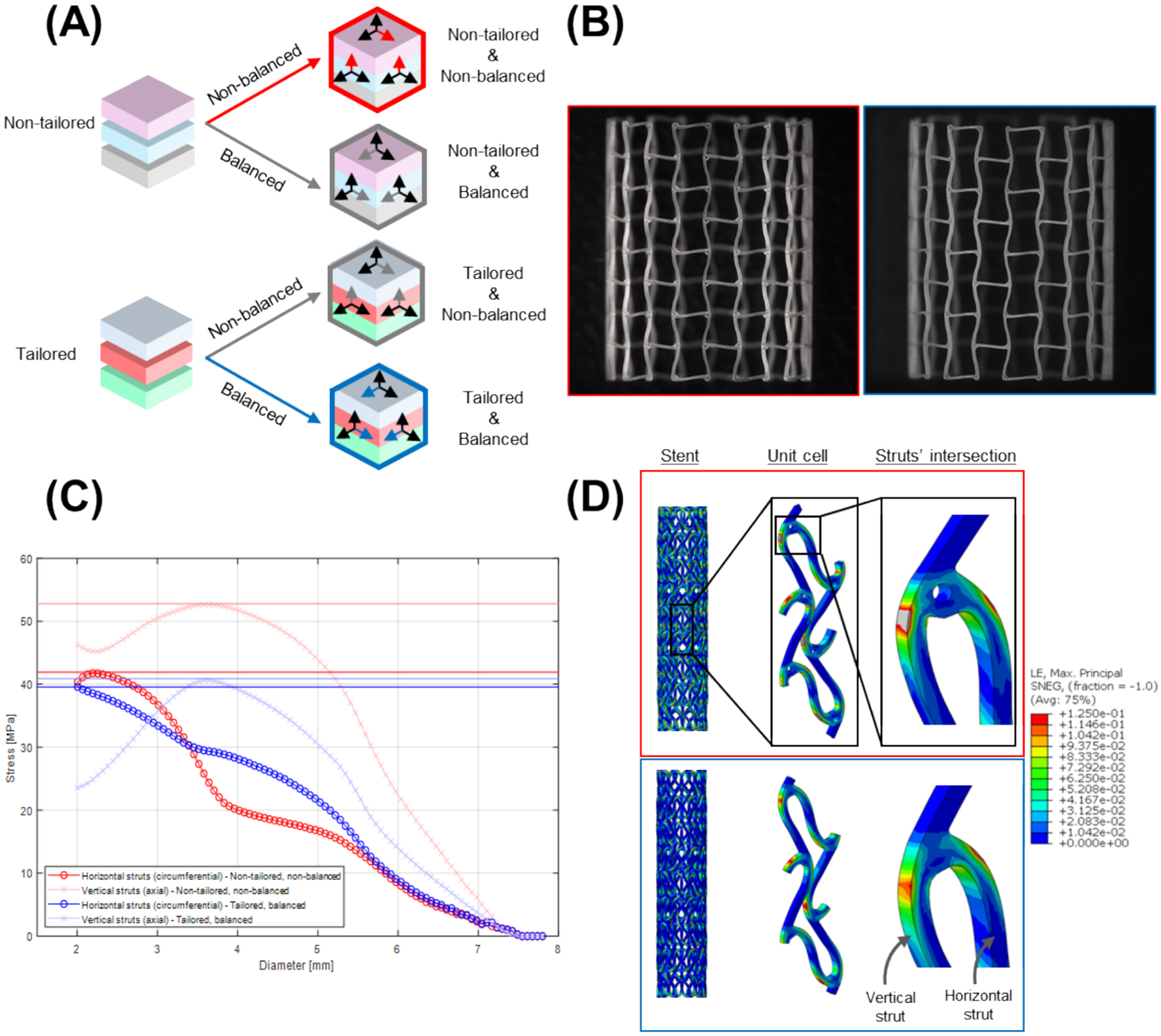
(A) Stent configurations resulting from the combination of two as-manufactured material characteristics and implantation-related stress distributions. (B) Microscope image of stent designs. (C) FEA analysis of vertical and horizontal struts’ average stress; S_max-NT-NB-horizontal_ = 41.91 MPa, S_max-NT-NB-vertical_ = 52.78 MPa, S_max-T-B-horizontal_ = 39.58 MPa, S_max-T-B-vertical_ = 40.89 MPa. (D) FEA analysis of logarithmic strain in critical stress region (intersection between vertical and horizontal struts). Red frame: non-tailored, non-balanced design. Blue frame: tailored, balanced design.

This behavior was achieved by fine-tuning the stiffness and geometry of the critical regions in both strut types so that the maximum stress in the vertical and horizontal struts did not differ excessively. As a result, the balanced design displayed lower peak stresses and a more uniform strain distribution across the stent. Additionally, the average radial force after expansion to diameters between 4.5 and 5.5 mm was 0.1457 ± 0.0057 N/mm for the non-tailored, non-balanced design, compared to 0.1958 ± 0.0029 N/mm for the tailored, balanced design. These mechanical differences were evident in the simulation results shown in Fig. 6C and D.

**Fig. 7.**
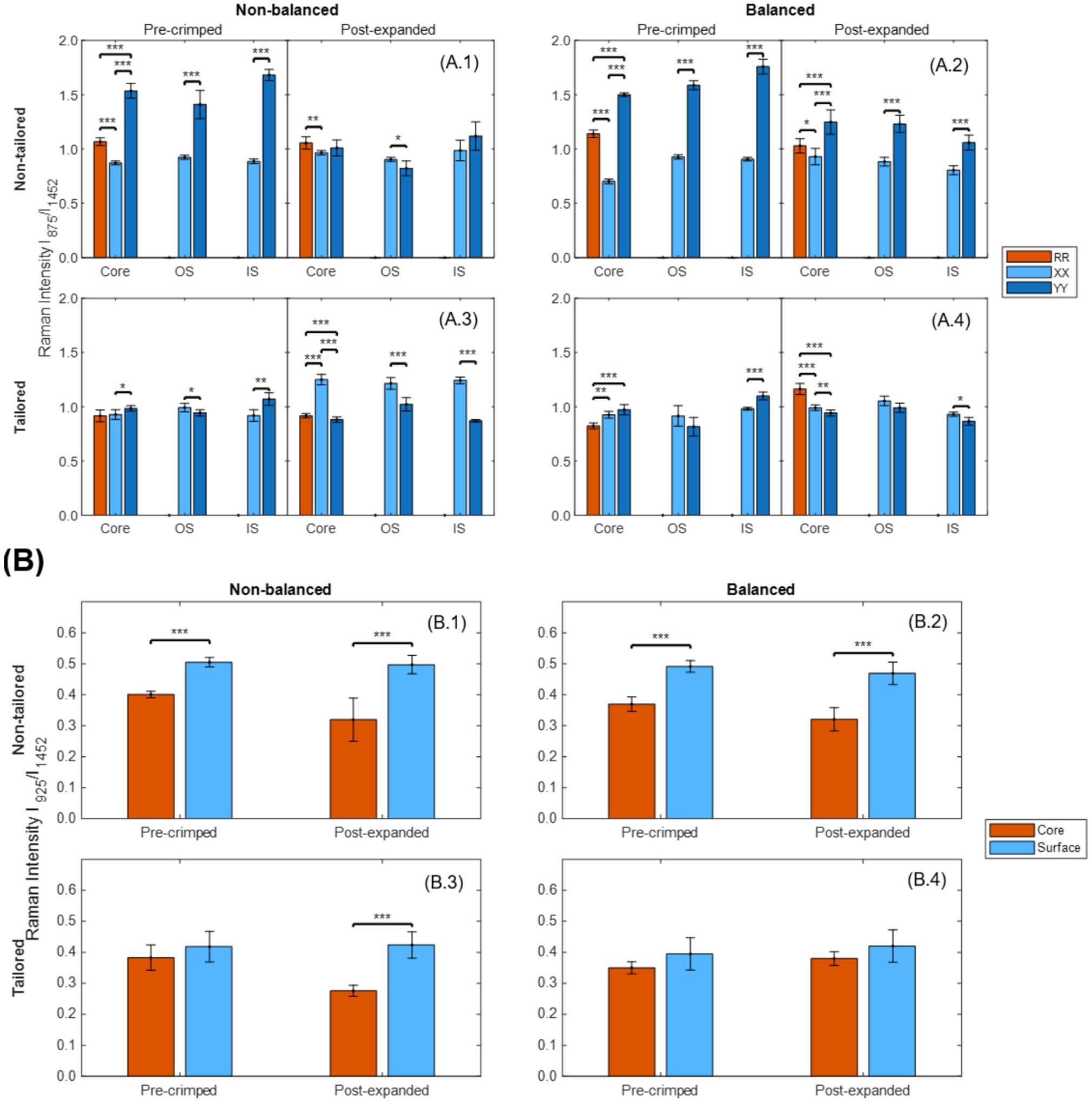
(A) Polarized and (B) non-polarized Raman spectroscopy intensity ratios for each pre-crimped and post-expanded stent configuration. (A.1, B.1) Non-tailored, non-balanced; (A.2, B.2) Non-tailored, balanced; (A.3, B.3) Tailored, non-balanced; (A.4, B.4) Tailored, balanced.

The macroscopic properties of each stent configuration—determined by its specific tubing and design—stemmed from distinct microstructural characteristics. All BRS were analyzed via Raman spectroscopy to assess microstructural ordering both post-manufacturing and after implantation, as illustrated in Fig. 7. Notably, as the polymer chains grow longer and align predominantly in a single direction, stretching the extended backbone enhances the response to the incident polarized light, leading to a stronger I_875_ signal. The CH_3_ rocking mode mixed with the stretch mode of the backbone is associated with the crystalline phase of the polymer, such that higher crystallinity results in increased I_925_. I_11,425_ is taken as an internal normalization standard [26].

For the non-tailored, non-balanced stent, pre-crimped polarized data showed stronger polymer chain alignment in the circumferential direction throughout the stent. After expansion, this alignment shifted: circumferential alignment remained predominant in the core and inner surface layers, while the outer surface layer showed a shift toward axial alignment, reflecting reduced circumferential ordering. Crystallinity was higher at the surface than in the core before crimping, with this disparity increasing following implantation.

For the non-tailored, balanced stent, pre-crimped polarized Raman spectra indicated clear circumferential polymer chain alignment across all layers. Post-expansion, this orientation persisted, though to a lesser extent, maintaining predominant circumferential alignment. Crystallinity remained higher at the surface than the core in both pre-crimped and post-expanded states.

For the tailored, non-balanced stent, pre-crimped data showed predominant circumferential alignment in the core and inner surface layers, but axial alignment in the outer surface layer, indicating no dominant overall polymer orientation. After expansion, the alignment shifted toward the axial direction across all layers. Crystallinity was uniform between surface and core before crimping but diverged notably after expansion, being higher in the surface than in the core.

For the tailored, balanced stent, pre-crimped data showed mixed polymer chain orientations across layers, with no consistent alignment pattern. Post-expansion, minimal disruption was observed, with polymer chains tending toward axial alignment in most layers. Crystallinity remained stable between surface and core across both pre-crimped and post-expanded states, reflecting microstructural stability during implantation. Numerical data are provided in Supplementary Table S1.

### 3.2. Microstructural properties of the As-Manufactured stents

**Fig. 8.**
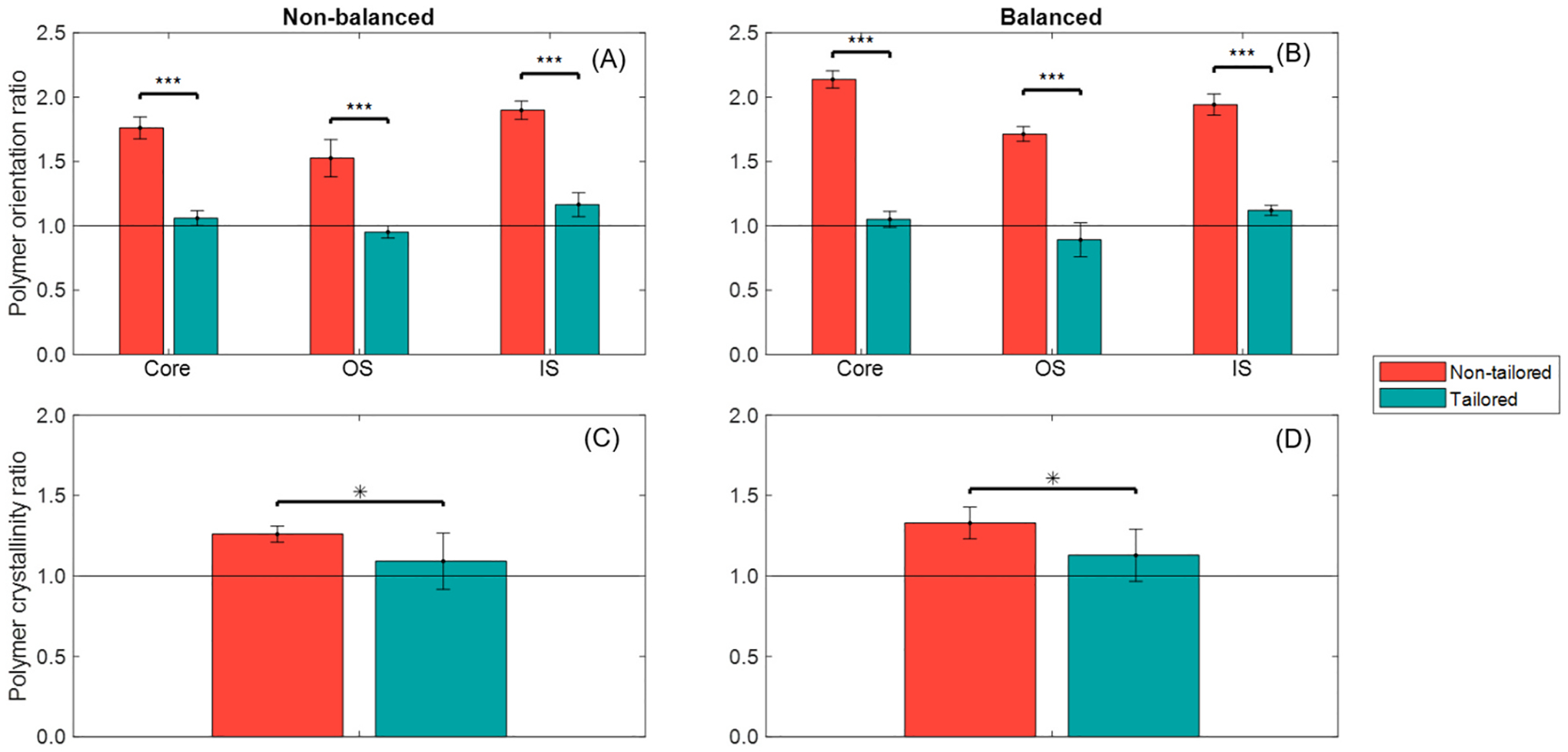
Fabrication process’ strain induced impact on the microstructural ordering of the BRS: Polymer orientation and crystallinity ratios for each as-manufactured stent configuration. (A) Polymer orientation ratio, non-balanced; (B) Polymer orientation ratio, balanced; (C) Polymer crystallinity ratio, non-balanced; (D) Polymer crystallinity ratio, balanced.

Raman intensity ratios of as-manufactured stents revealed polymer orientation and crystallinity differences between non-tailored and tailored tubing, as shown in Fig. 8. Polymer orientation ratios confirmed that non-tailored stents exhibited a pronounced circumferential alignment, while tailored stents showed a more uniform alignment between axial and circumferential directions. Both stent types demonstrated consistent polymer alignment across the stent layers. Polymer crystallinity ratios indicated higher surface crystallinity in non-tailored stents, whereas tailored stents displayed a more homogeneous crystallinity distribution between surface and core. Numerical data are provided in Supplementary Table S2.

### 3.3. Implantation process impact on the microstructural properties of stents

**Fig. 9.**
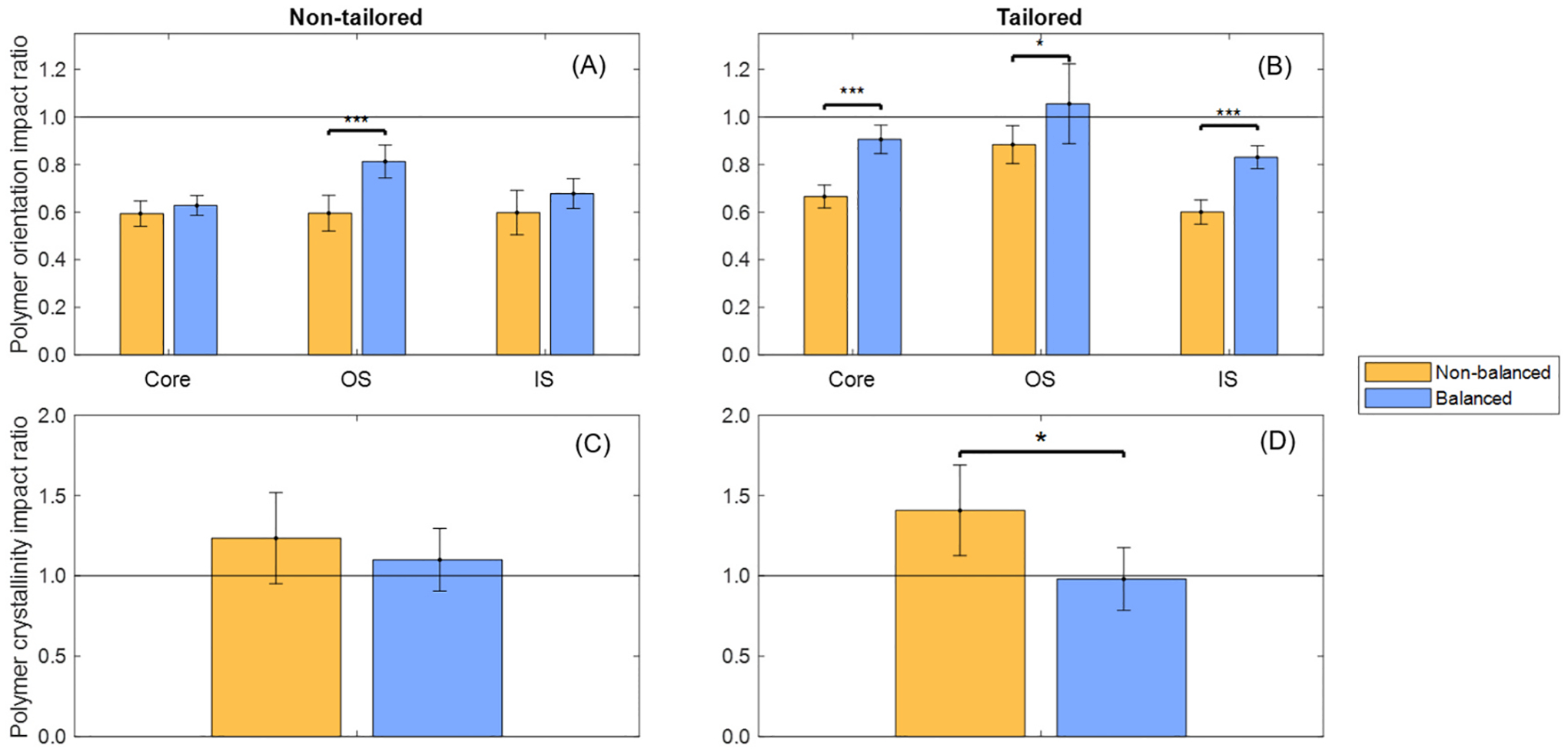
Implantation process’ strain-induced impact on the microstructural ordering of the BRS: Polymer orientation and crystallinity impact ratios for each stent configuration. (A) Polymer orientation impact ratio, non-tailored; (B) Polymer orientation impact ratio, tailored; (C) Polymer crystallinity impact ratio, non-tailored; (D) Polymer crystallinity impact ratio, tailored.

Data processing of polymer orientation and crystallinity ratios from pre-crimped and post-expanded stents yielded impact ratios that highlight how implantation-related strain affects the as-manufactured microstructural ordering of each stent configuration, as shown in Fig. 9.

The non-tailored stents initially exhibited polymer chains predominantly aligned in the circumferential direction, but this microstructural ordering was affected up to a 41% extent by implantation in both the non-balanced and balanced stent designs. The tailored stents started with a more uniform polymer alignment between axial and circumferential directions. Implantation of the non-balanced stent caused a shift of polymer chains up to a 33% extent toward the axial direction, whereas the balanced design minimized this shift to a 17% extent, maintaining more consistent polymer alignment throughout the stent.

Analysis of crystallinity impact showed no significant differences between the non-balanced and balanced designs in non-tailored stents. In contrast, for tailored stents, the balanced design preserved uniform crystallinity with minimal disruption across stent layers. Numerical data are provided in Supplementary Table S3.

### 3.4. Degradation behavior

**Fig. 10.**
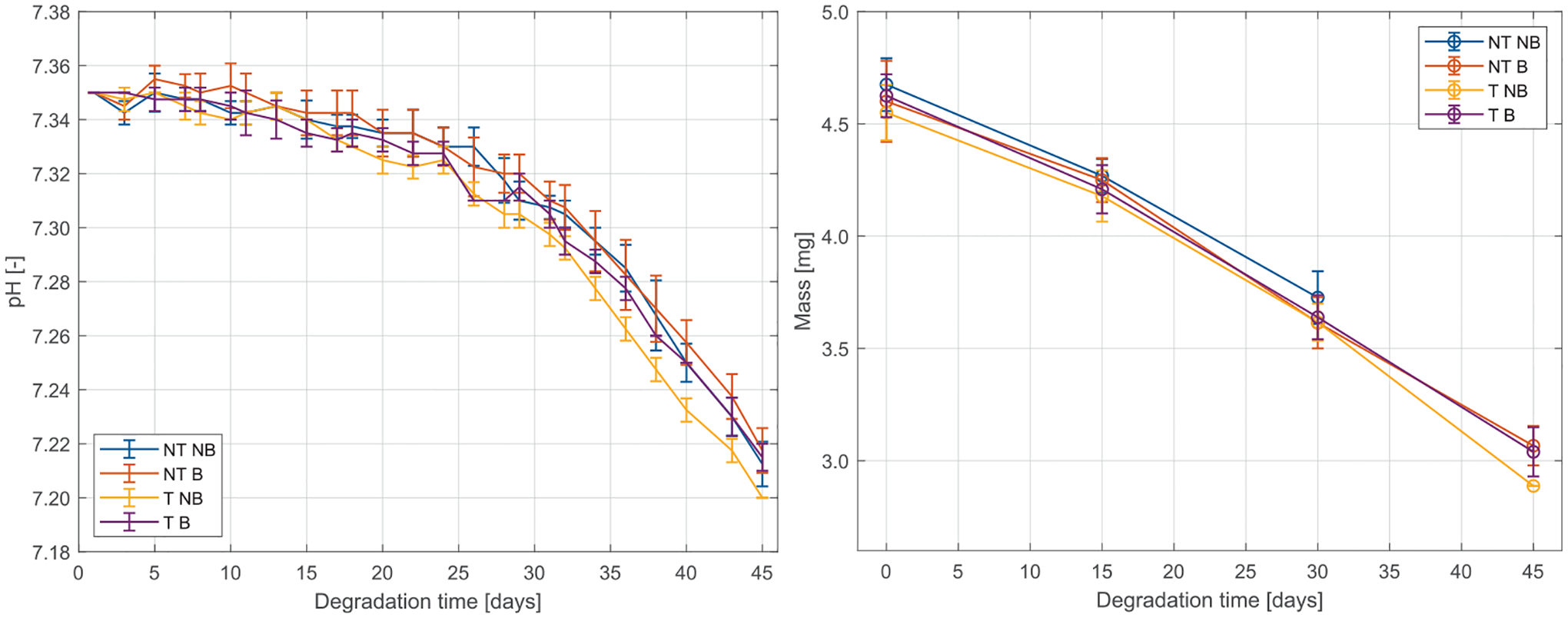
Quantitative assessment of each PLCL stent configuration’s degradation from 0 to 45 days of accelerated thermal degradation. Left: PBS degradation media’s pH progression. Right: stent’s mass progression.

The implanted BRS degraded consistently with the accelerated thermal model [39]. The PBS in contact with PLCL stents showed acidification, starting at pH 7.35 on day zero of accelerated degradation. The PBS solution in contact with the PLCL stents acidified, starting at a pH of 7.35 on day zero and dropping to 7.20 after 45 days under accelerated conditions. This pH decrease followed an exponential trend, indicating accelerated degradation during the later stages of the experiment (Fig. 10 – Left). Mass loss mirrored this pattern (Fig. 10 – Right). By day 45, non-tailored, non-balanced samples degraded to the extent that mass measurement was not possible, while tailored, non-balanced stents fragmented fully but left measurable remnants. Both balanced stent configurations retained enough structural integrity for accurate mass assessment.

PLCL stents showed a mass loss of 8.37 ± 0.61% by day 15, increasing to 20.89 ± 0.57% at 30 days, and 34.72 ± 1.65% by day 45. The degradation rate increased over time: from day 0 to 15, mass loss occurred at 0.59 ± 0.04% per day, rising to 0.84 ± 0.06% per day from day 15 to 30, and peaking at 0.92 ± 0.13% per day from day 30 to 45.

**Fig. 11.**
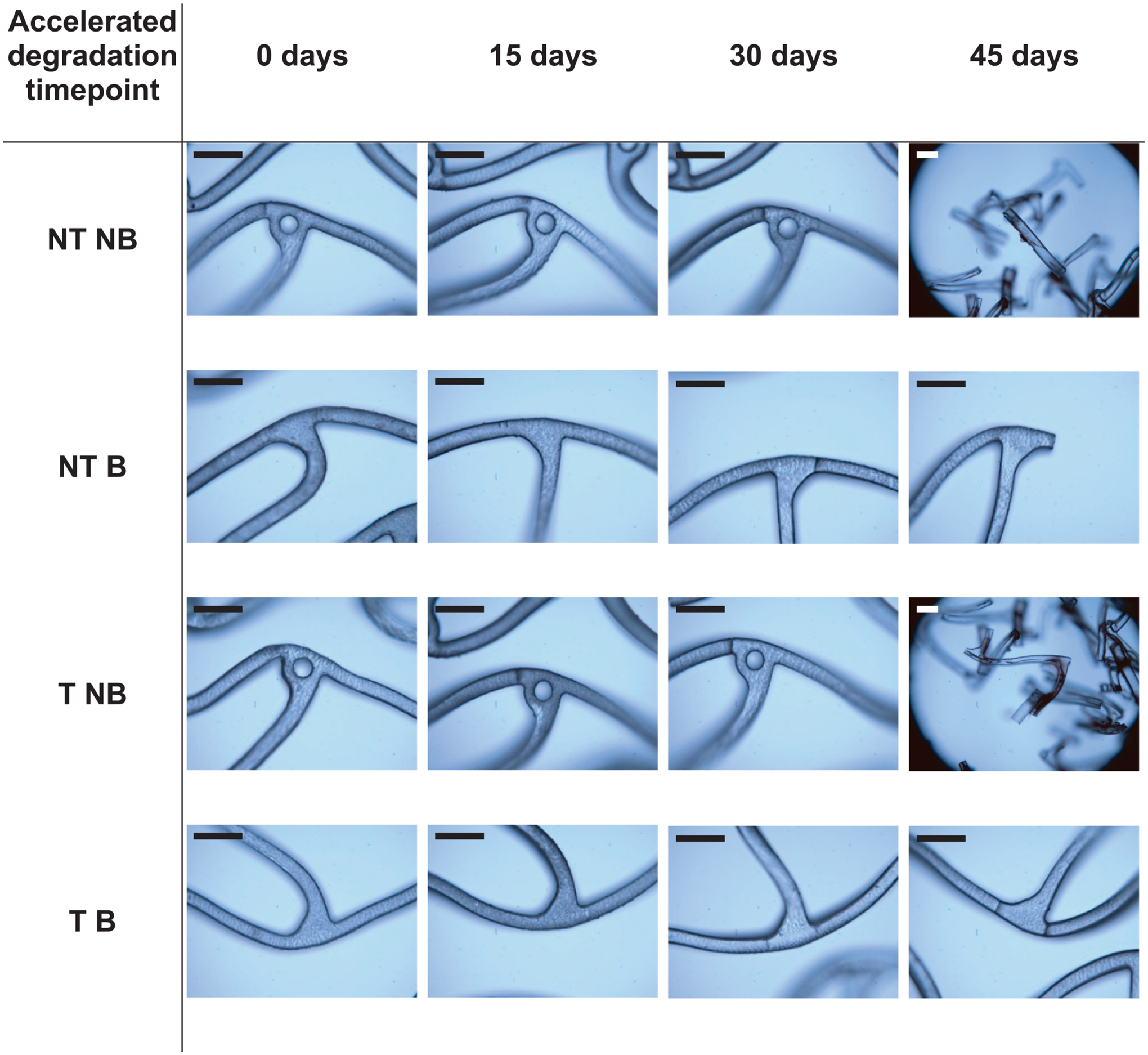
Confocal microscope images of each PLCL stent configuration at 0, 15, 30 and 45 days of accelerated thermal degradation. Focus on high-stress regions presenting enhanced localized deformation and microcrack formation. Scale bar = 300 μm.

Degradation of the different stent configurations was further evaluated by confocal microscopy, targeting regions exhibiting notable loss of structural integrity, as illustrated in Fig. 11.

Immediately after implantation, no degradation was detected, but high-strain regions were apparent in certain configurations. Non-balanced stents exhibited dimples and microcracks, indicating uneven degradation, whereas balanced designs showed minimal microcracking and reduced strain in critical areas. After 15 days of accelerated degradation, structural integrity remained largely unchanged from day zero; non-balanced stents continued to display microcracks, while balanced, non-tailored stents showed only subtle disruptions. Tailored, balanced stents degraded uniformly without structural damage or increased resorption. By 30 days, non-tailored and tailored, non-balanced stents experienced significant localized degradation, characterized by cracks and fragmented struts. Tailored, balanced stents exhibited less disruption, with only minor cracks observed. At the conclusion of the thermal degradation study, all samples presented substantial damage: non-balanced stents were fully fragmented, non-tailored, balanced stents had broken struts, and tailored, balanced stents maintained the best structural integrity despite notable damage.

### 3.5. Thrombogenicity behavior

All BRS-implanted loops, metallic stent-implanted loops, and negative controls were filled with human blood and exposed to physiological flow conditions.

**Fig. 12.**
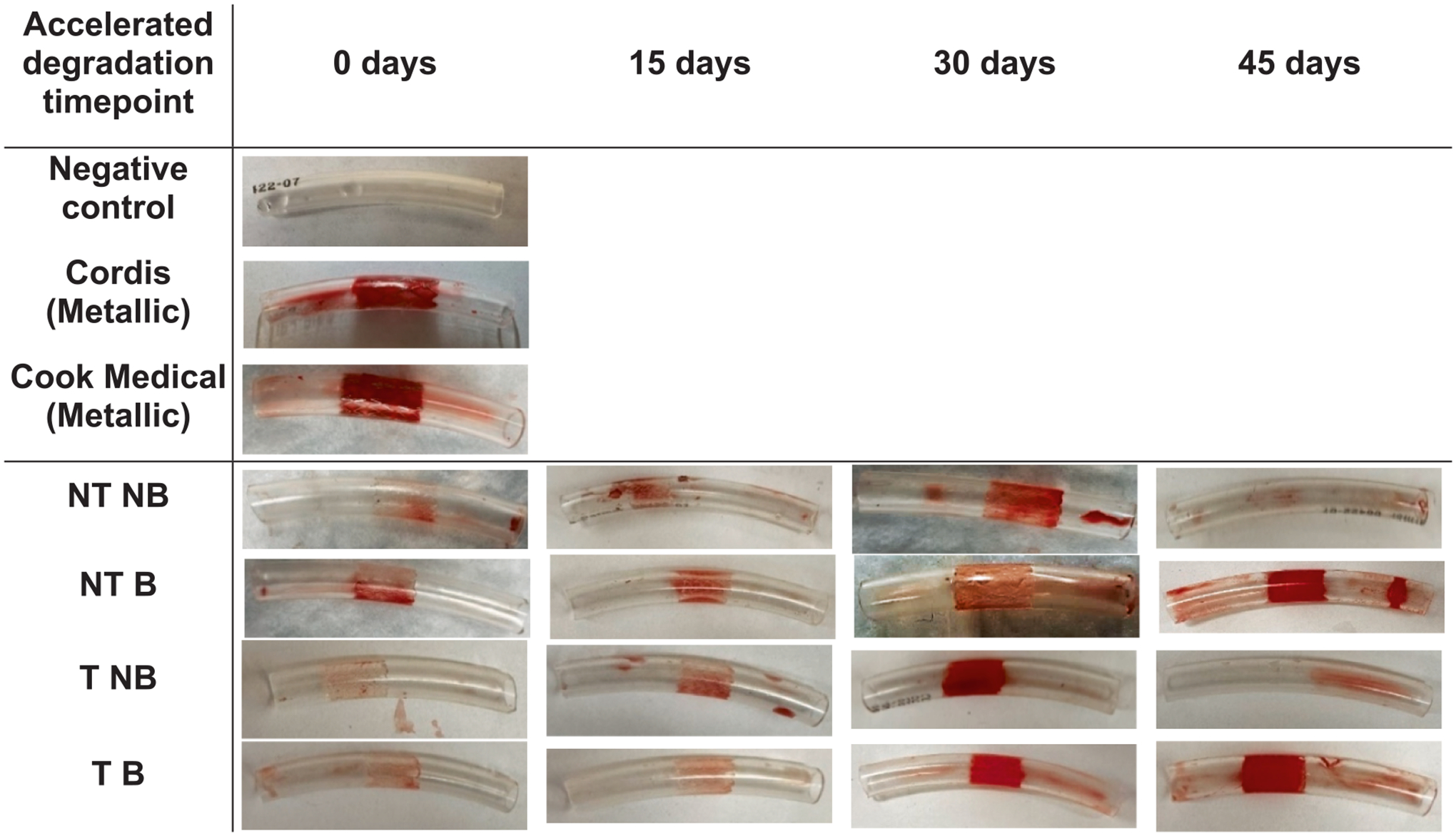
Visual assessment of blood clot formation in reactive segments of negative controls, metallic stents, and polymeric stents at different degradation timepoints.

Images of the reactive segments revealed distinct thrombus formation patterns (Fig. 12). Negative controls showed minimal clotting, while metallic stents exhibited thrombus accumulation at the implantation sites. Polymeric stents demonstrated progressively increased clot formation as degradation advanced. Moreover, as the stents degraded, their structural integrity weakened, leading to fragmentation and release of small debris into the bloodstream.

The stents’ structural integrity correlated directly with the number of stents remaining intact. At 30 days of accelerated degradation, one tailored, non-balanced stent fragmented. By 45 days, fragmentation was observed in three non-tailored, non-balanced stents, two non-tailored, balanced stents, and three tailored, non-balanced stents. Only the tailored, balanced stents consistently withstood physiological blood flow without breaking apart.

**Fig. 13.**
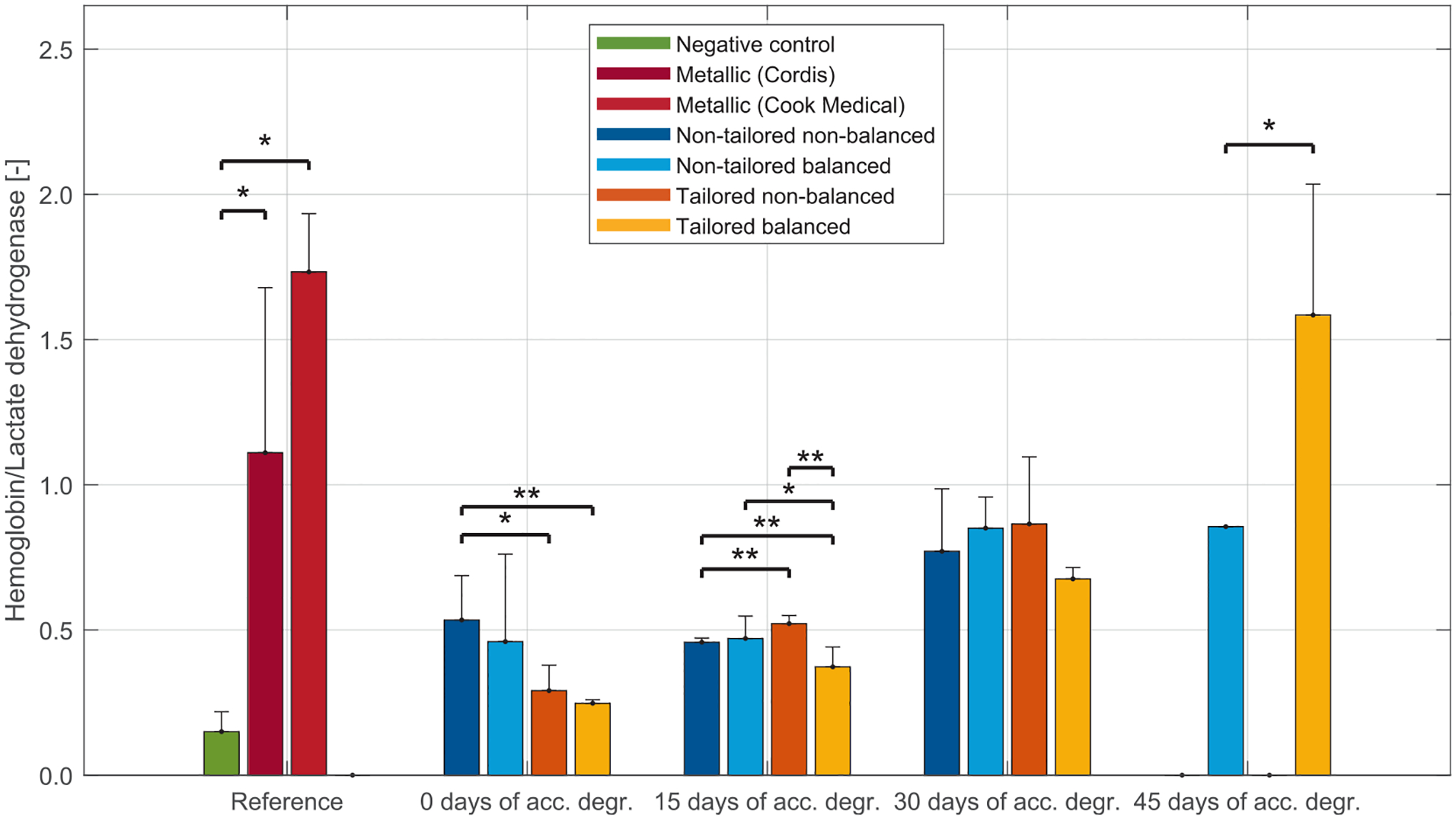
HGB/LDH quantitative assessment of blood clot formation in reactive segments of negative controls, metallic stents, and polymeric stents at different degradation timepoints.

Metallic stents exhibited consistently high HGB/LDH values, with Cordis Genesis recording 1.11 ± 0.57 and Cook Medical Formula reaching 1.73 ± 0.20. In contrast, polymeric stents showed a broader spectrum of coagulation responses, ranging from 0.25 ± 0.01 for the tailored, balanced design immediately after implantation to 1.58 ± 0.45 for the same configuration following 45 days of thermal degradation, as shown in Fig. 13.

Immediately post-implantation, clot formation varied across polymeric stents, with tailored designs generating less thrombosis than the non-tailored, non-balanced stents. At 15 days, differences among configurations were minimal, though the tailored, balanced stents remained slightly less thrombogenic. This tendency persisted through 30 days, albeit without statistical significance. By 45 days, the tailored, balanced stents were the only configuration where all samples maintained resistance to physiological flow. However, structural degradation at this stage led to increased thrombus formation. Additional data obtained by pooling the results according to stent configuration and degradation timepoint are provided in the Supplementary Fig. S2 and Fig. S3.

## 4. Discussion

Studies have long shown that material properties and device design dominate stents’ mechanical performance [40,41]. By systematically combining two polymer tubing formations (non-tailored vs. tailored, Fig. 5) and two stent geometries (non-balanced vs. balanced, Fig. 6), we highlighted the interplay between material and design characteristics and identified key conception rules for next-generation BRS.

The specific configurations analyzed exhibited mild radial force values, within the same order of magnitude as those reported for investigational pediatric BRS in the literature (0.19–0.32 N/mm for a strut thickness of 250 μm) [42]. These properties may be particularly suitable for pediatric vascular indications where the stent functions primarily as a complementary scaffold after balloon angioplasty [43]. In these settings, angioplasty achieves luminal gain by disrupting fibrotic fibers, with limited tissue recoil reducing the need for sustained high radial force [44,45]. Material selection is central to achieving this balance: PLCL was chosen as a more elastic alternative to PLLA, minimizing the risk of fracture while also contributing to reduced device–artery compliance mismatch in pediatric vessels [39,46]. Moreover, PLCL has been reported to exhibit reduced platelet adhesion compared to PLLA, further supporting its suitability for vascular applications [47].

In semi-crystalline polymers like PCL or PLLA, the microstructural properties, such as polymer chain alignment and crystallinity, are critical for mechanical performance [14,48]. For example, solid-phase die drawing of PLLA tubing aligns polymer chains and dramatically increases tensile modulus (+79.7%), yield strength (+121.5%) and ultimate strength (+267.9%) relative to as-extruded material. This alignment also generates higher crystallinity (~40% vs ~ 1.3%), which imparts greater radial stiffness to laser-cut stents [49]. In this study, the non-tailored tubing imparts strong circumferential alignment and non-uniform crystallinity (Fig. 8). Under implantation, crimping and balloon expansion (Fig. 9) disrupt this ordering: stress-mismatched configurations develop regions of chain misorientation and local amorphization, which may compromise clinical performance reliability. In contrast, the tailored tubing begins with balanced alignment and homogeneous crystallinity; when employed in the balanced design, it preserves microstructural ordering through deployment, minimizing local heterogeneity and fostering predictable mechanical response [26].

In turn, these microstructural features modulate degradation: crystallized regions hydrolyze more slowly, whereas amorphous regions are more prone to chain scission and autocatalytic breakdown. Experimental studies show that these effects, along with heterogeneous degradation, significantly alter a polymer’s mechanical properties over time [50]. In addition, adding elastic PCL segments to PLLA markedly accelerates overall hydrolysis and changes crystallization behavior [51]. In this study, implanted BRS undergo hydrolytic degradation that further modulates function beyond initial microstructural properties. All configurations exhibited progressive mass loss and pH drop in PBS—signatures of amorphous region hydrolysis—and became increasingly brittle. Non-tailored, and tailored, non-balanced stents, already burdened by stress-induced microstructural heterogeneity, developed cracks and fragmentations in high-strain zones due to accelerated hydrolysis. In contrast, tailored, balanced stents degraded more uniformly, sustaining structural integrity longer. These findings underscore that tailoring the polymer microstructure is a powerful way to optimize stent lifespan. By proactively aligning polymer orientation with the predicted stress field, our study directly addresses the stress-degradation coupling. Prior stents have largely ignored this “stress map”: reference BRS (e.g. Absorb [52] or DESolve [53]) present thin “hinge” regions that crack as the material degrades under cyclic load. Tailoring the microstructure to stress could prevent such focal failures and better preserve mechanical performance where the vessel needs it.

Degradation directly impacts hemodynamic performance, a major clinical concern with BRS [54]. The Abbott Absorb stent (150–170 μm PLLA struts), which fully resorbs by ~ 3 years, presented device thrombosis that was 2.4 % vs 0.6 % for Xience (metal everolimus-eluting stent) [55] and platelet coverage that was 21.8 % vs 3.0 % for Magmaris (biocorrodible magnesium stent) [56–58]. These trends imply that polymeric stents – especially as they begin to degrade and leave behind fibrin-laden grooves – pose a greater risk of clot formation than comparably-dimensioned metallic stents. In this study, polymeric stents induced significantly less clot formation than metallic controls during the initial stages after implantation. However, beyond this initial stage—when degradation became pronounced—thrombogenicity rose exponentially, approaching levels observed with metal stents. The tailored, balanced design maintained the lowest thrombosis risk throughout, thanks to its preserved microstructure and structural cohesion. Yet even these stents, once highly degraded, matched the clotting propensity of metallic devices, highlighting that structural integrity alone cannot curb thrombosis indefinitely.

Although the present study held surface processing constant to focus on microstructural effects, it is acknowledged that absolute surface roughness induced by laser cutting, as well as polymer functionalization, can further increase thrombogenic potential *in vivo* [59,60]. To reduce this confounder, all polymeric stents were fabricated under identical femtosecond laser-cutting conditions to minimize between-group variability. Nevertheless, dedicated analyses of surface properties and their effects on platelet adhesion and thrombus formation are necessary for full clinical translation.

Notably, polymeric stents must balance adequate radial support at low wall thickness with resistance to premature failure — a balance that strongly relies of microstructure. Minimizing localized strain gradients preserves polymer crystallinity and chain integrity, maintaining effective material strength [26,61]. The balanced, tailored design compensates stiffness in critical regions to produce a more uniform strain field; which in turn yielded higher radial force after expansion. Thus, reducing microstructural disruption complements geometric optimization to sustain both immediate performance and long-term structural lifespan. Yet, therapeutic efficacy requires the proper adjustment of stent mechanical properties, such as radial strength and flexibility, to the specific requirements of the intended application (vessel size, lesion type, delivery route, etc.).

These observations motivate a two-pronged development strategy. First, one can predict the post-implantation microstructure from known “inputs”—the as-manufactured polymer alignment and crystallinity, and the stress field computed by FEA. Larger mismatches between these inputs lead to greater microstructural heterogeneity in the deployed stent. Second, one can refine the inputs—tune extrusion and expansion parameters to tailor polymer microstructure, and adjust strut geometry to shape stress distribution—to minimize this mismatch. In practice, a reverse-engineering workflow begins with the target vessel and lesion, derives the necessary stent geometry and implantation stresses via modeling, and then custom-fabricates tubing whose microstructure is engineered to match those stresses. This approach ensures that macroscopic mechanics and microstructural ordering are inherently aligned. This development strategy benefits the interval during which thrombogenicity remains low before degradation-driven clotting elevates clinical risks. However, rapid and robust tissue integration must occur before structural breakdown to seal the blood–polymer interface, preventing fragment embolization and reducing thrombogenicity at later stages.

Optimizing BRS performance demands a holistic integration of material processing, stent geometry, degradation kinetics, and biological response. By engineering polymer microstructure to match implantation stresses, and by fostering early tissue growth, we can create stents that not only perform mechanically upon implantation but also maintain prolonged functionality throughout their lifespan.

## 5. Conclusions

Concern persists that polymeric stent thrombosis is an inherent trade-off for reducing complications associated with permanent metallic implants. However, suboptimal outcomes with BRS stem more from design and evaluation oversights than inherent limitations. Macroscopic performance depends on post-implantation microstructural properties. Yet, existing literature has overlooked how localized disruptions directly affecting performance vary with the interaction between microstructural properties and applied stress vectors. Our findings demonstrate that the extent of disruption depends not merely on the magnitude of stress, but critically on the degree of misalignment between polymer chain orientation and stress direction.

Our study showed that achieving minimized disruption in polymeric chains alignment and uniform crystallinity across layers requires harmonized interaction between the microstructural ordering of the as-manufactured device and the implantation-related stress distribution of the design. Reducing the mismatch between these features is as critical as adjusting their properties to the stent’s intended application. Greater uniformity across regions enables sustained and controlled crack formation during degradation, which extends the structural lifespan of BRS—a critical factor for clinical safety, as the risk of thrombosis rises sharply well before polymer fragmentation occurs. This study demonstrated that polymer tubing and stent design must be developed synergistically to deliver a device that overcomes current BRS limitations.

## Supplementary Material

1

## Data Availability

Data will be made available on request.

## Appendix A. Supplementary data

Supplementary data to this article can be found online at https://doi.org/10.1016/j.matdes.2025.115013.
