# Supplementary material for: Integrating stent design and microstructural characterization to improve clinical outcomes of bioresorbable stents
Source: Mater Des. Author manuscript; Available in PMC 2025 Dec 31. (PMC12753002; doi:10.1016/j.matdes.2025.115013)
Supplement: 1 [file NIHMS2132183-supplement-1.docx]

# Supplementary Material


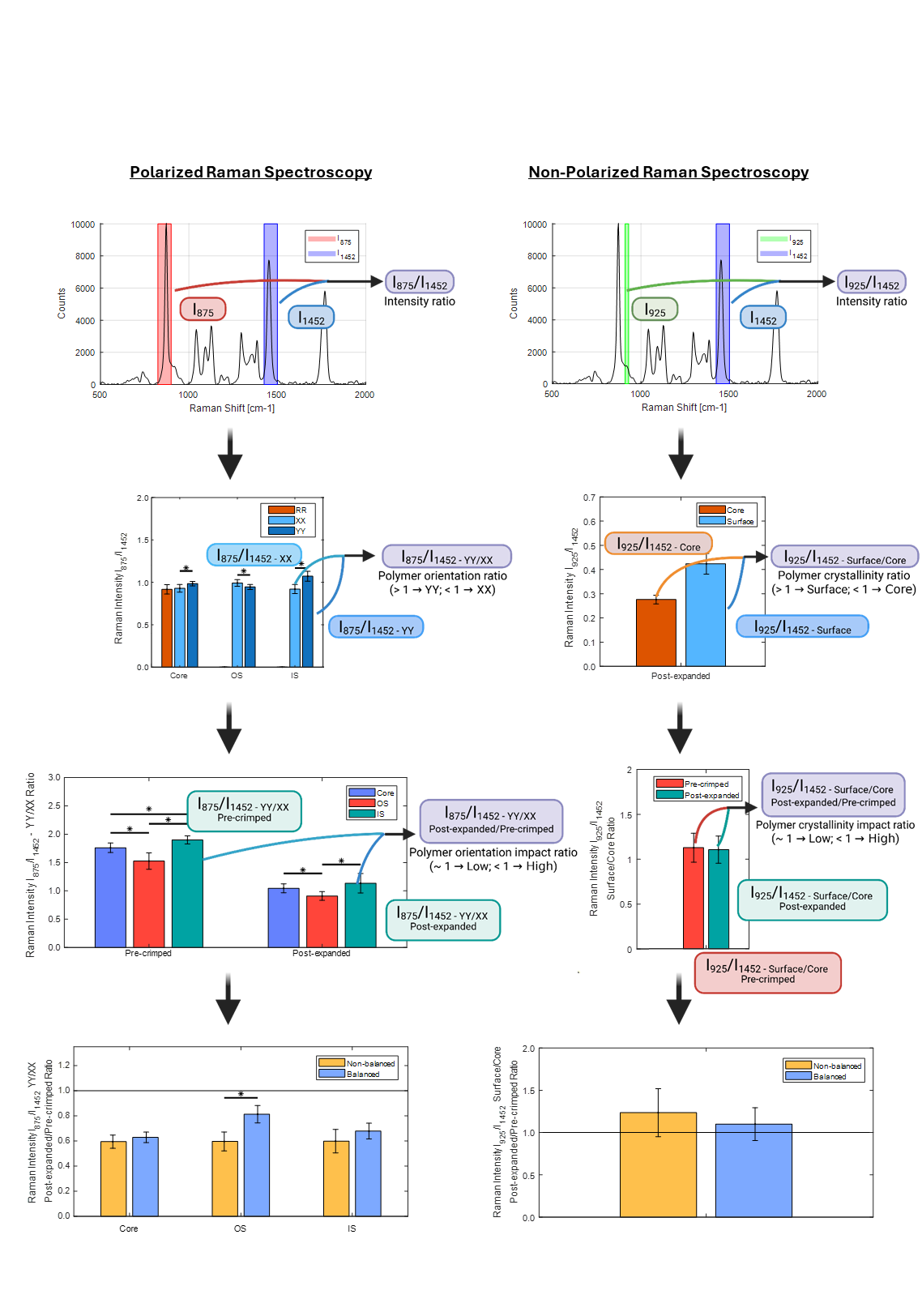


Figure S 1. detailed schematic representation of the Raman spectroscopy data analysis process and ratio calculations (specter 🡪 intensity ratios 🡪 orientation ratios and crystallinity ratios 🡪 implantation ratios)

Table S 1. Detail of polarized and non-polarized Raman spectroscopy microstructural characterization results for each pre-crimped and post-expanded stent configuration. Polarized results are presented as I_yy_ vs. I_xx_. Non-polarized results are presented as I_Surface_ vs. I_Core_. [*] indicates no statistical significance (p = 0.05).

|  |  | **Non-tailored, non-balanced** | | **Non-tailored, balanced** | | **Tailored, non-balanced** | | **Tailored, balanced** | |
| --- | --- | --- | --- | --- | --- | --- | --- | --- | --- |
|  |  | *Pre-crimping* | *Post-expansion* | *Pre-crimping* | *Post-expansion* | *Pre-crimping* | *Post-expansion* | *Pre-crimping* | *Post-expansion* |
| **Polarized intensity ratios** | *Core* | I_yy_ > I_xx_  1.54 ± 0.07  >  0.87 ± 0.02 | I_yy_ >* I_xx_  1.01 ± 0.07  >*  0.97 ± 0.02 | I_yy_ > I_xx_  1.50 ± 0.02  >  0.70 ± 0.02 | I_yy_ > I_xx_  1.25 ± 0.11  >  0.93 ± 0.08 | I_yy_ > I_xx_  0.98 ± 0.03  >  0.93 ± 0.04 | I_yy_ < I_xx_  0.70 ± 0.02  <  1.25 ± 0.05 | I_yy_ >* I_xx_  0.97 ± 0.05  >*  0.93 ± 0.03 | I_yy_ < I_xx_  0.94 ± 0.03  <  0.99 ± 0.03 |
|  | *OS* | I_yy_ > I_xx_  1.41 ± 0.13  >  0.92 ± 0.02 | I_yy_ < I_xx_  0.82 ± 0.07  <  0.90 ± 0.02 | I_yy_ > I_xx_  1.59 ± 0.04  >  0.93 ± 0.02 | I_yy_ > I_xx_  1.23 ± 0.08  >  0.88 ± 0.04 | I_yy_ < I_xx_  0.94 ± 0.03  <  0.99 ± 0.04 | I_yy_ < I_xx_  0.22 ± 0.05  <  1.22 ± 0.05 | I_yy_ <* I_xx_  0.82 ± 0.09  <*  0.92 ± 0.09 | I_yy_ <* I_xx_  0.99 ± 0.04  <*  1.05 ± 0.04 |
|  | *IS* | I_yy_ > I_xx_  1.68 ± 0.05  >  0.89 ± 0.02 | I_yy_ >* I_xx_  1.12 ± 0.13  >*  0.99 ± 0.09 | I_yy_ > I_xx_  1.76 ± 0.07  >  0.91 ± 0.02 | I_yy_ > I_xx_  1.06 ± 0.07  >  0.80 ± 0.04 | I_yy_ > I_xx_  1.07 ± 0.06  >  0.92 ± 0.05 | I_yy_ < I_xx_  0.87 ± 0.01  <  1.24 ± 0.30 | I_yy_ > I_xx_  1.10 ± 0.04  >  0.98 ± 0.01 | I_yy_ < I_xx_  0.87 ± 0.04  <  0.93 ± 0.02 |
| **Non-polarized intensity ratios** | | I_Surface_ > I_Core_  0.50 ± 0.02  >  0.40 ± 0.01 | I_Surface_ > I_Core_  0.50 ± 0.03  >  0.32 ± 0.07 | I_Surface_ > I_Core_  0.49 ± 0.02  >  0.37 ± 0.02 | I_Surface_ > I_Core_  0.47 ± 0.04  >  0.32 ± 0.04 | I_Surface_ >* I_Core_  0.42 ± 0.05  >*  0.38 ± 0.04 | I_Surface_ > I_Core_  0.42 ± 0.04  >  0.28 ± 0.02 | I_Surface_ >* I_Core_  0.39 ± 0.05  >*  0.35 ± 0.02 | I_Surface_ >* I_Core_  0.42 ± 0.05  >*  0.38 ± 0.02 |

Table S 2. Detail of polymer orientation and crystallinity ratios for each as-manufactured stent configuration. Polymer orientation ratio results are presented as POR_-Non-tailored_ vs. POR_-Tailored_. Polymer crystallinity ratio results are presented as PCR_-Non-tailored_ vs. PCR_-Tailored_.

|  |  | **Non-balanced** | **Balanced** |
| --- | --- | --- | --- |
| **Polymer orientation ratio** | *Core* | POR_-Non-tailored_ > POR_-Tailored_  1.76 ± 0.09 > 1.06 ± 0.06 | POR_-Non-tailored_ > POR_-Tailored_  1.71 ± 0.06 > 0.89 ± 0.01 |
|  | *OS* | POR_-Non-tailored_ > POR_-Tailored_  1.53 ± 0.14 > 0.95 ± 0.05 | POR_-Non-tailored_ > POR_-Tailored_  1.76 ± 0.03 > 0.99 ± 0.03 |
|  | *IS* | POR_-Non-tailored_ > POR_-Tailored_  2.14 ± 0.07 > 1.05 ± 0.06 | POR_-Non-tailored_ > POR_-Tailored_  1.94 ± 0.08 > 1.12 ± 0.04 |
| **Polymer crystallinity ratio** | | PCR_-Non-tailored_ > PCR_-Tailored_  1.26 ± 0.05 > 1.09 ± 0.18 | PCR_-Non-tailored_ > PCR_-Tailored_  1.33 ± 0.10 > 1.13 ± 0.16 |

Table S 3. Detail of polymer orientation and crystallinity impact ratios for each stent configuration. Polymer orientation impact ratio results are presented as POIR_-Non-tailored_ vs. POIR_-Tailored_. Polymer crystallinity impact ratio results are presented as PCIR_-Non-tailored_ vs. PCIR_-Tailored._ [*] indicates no statistical significance (p = 0.05).

|  |  | **Non-tailored** | **Tailored** |
| --- | --- | --- | --- |
| **Polymer orientation impact ratio** | *Core* | POIR_-Non-balanced_ <* POIR_-Balanced_  0.59 ± 0.05 <* 0.63 ± 0.04 | POIR_-Non-balanced_ < POIR_-Balanced_  0.67 ± 0.05 < 0.91 ± 0.06 |
|  | *OS* | POIR_-Non-balanced_ < POIR_-Balanced_  0.60 ± 0.08 < 0.81 ± 0.07 | POIR_-Non-balanced_ < POIR_-Balanced_  0.88 ± 0.08 < 1.06 ± 0.17 |
|  | *IS* | POIR_-Non-balanced_ <* POIR_-Balanced_  0.60 ± 0.09 <* 0.68 ± 0.06 | POIR_-Non-balanced_ < POIR_-Balanced_  0.60 ± 0.05 < 0.83 ± 0.05 |
| **Polymer crystallinity impact ratio** | | PCIR_-Non-balanced_ >* PCIR_-Balanced_  1.23 ± 0.28 >* 1.10 ± 0.19 | PCIR_-Non-balanced_ > PCIR_-Balanced_  1.41 ± 0.28 > 0.98 ± 0.19 |

Figure S 2. HGB/LDH quantitative assessment of blood clot formation in reactive segments of pooled stent configurations. Conditions 𝑎, 𝑏, and c are statistically different. Samples were pooled by stent configuration at 0, 15, and 30 days of accelerated degradation, excluding data from day 45 due to inconsistent sample availability across configurations. Only non-fragmented stents were included in the analysis.

Figure S 3. HGB/LDH quantitative assessment of blood clot formation in reactive segments of pooled accelerated thermal degradation timepoints. Conditions 𝑎, 𝑏, c and d are statistically different. Samples were pooled by degradation timepoint to evaluate thrombogenicity trends over the course of accelerated thermal degradation, considering only non-fragmented stents in the analysis.
